# Development of Antisense Tools to Study *Bodo saltans* and Its Intracellular Symbiont

**DOI:** 10.1002/mbo3.70018

**Published:** 2025-04-23

**Authors:** Mastaneh Ahrar, Lorna Glenn, Marie Held, Andrew Jackson, Krzysztof Kus, Gregory D. D. Hurst, Ewa Chrostek

**Affiliations:** ^1^ Department of Evolution, Ecology and Behaviour, Institute of Infection, Veterinary and Ecological Sciences University of Liverpool Liverpool UK; ^2^ Centre for Cell Imaging University of Liverpool Liverpool UK; ^3^ Department of Infection Biology and Microbiomes, Institute of Infection, Veterinary and Ecological Sciences University of Liverpool Liverpool UK; ^4^ Department of Biochemistry University of Oxford Oxford UK; ^5^ Institute of Environmental Sciences, Faculty of Biology Jagiellonian University Krakow Poland

**Keywords:** aquatic symbiosis, gene silencing, molecular tools, obligate symbiosis, peptide nucleic acids

## Abstract

Obligate symbioses are common in nature and present a particular challenge for functional genetic analysis. In many cases, the host is a non‐model species with poor tools for genetic manipulation, and the symbiont cannot be cultured or its gene expression manipulated to investigate function. Here, we investigated the potential for using antisense inhibition to analyze host and symbiont gene function within an obligate aquatic symbiosis. We focused on the kinetoplastid host *Bodo saltans* and its bacterial symbiont, C*andidatus Bodocaedibacter vickermanii*, a member of Rickettsiales. We conclude that antisense inhibition is not feasible in the *Bodo saltans* and its symbiont, as the holobiont feeds on the antisense molecules—and increases in numbers—upon treatment with the antisense construct. Although our approach has proven unsuccessful, we have developed an array of protocols that can be used to study the biology of this microeukaryote and its microbial associates.

## Graphical abstract

Obligate symbioses present challenges for functional genetic analysis. This study shows that antisense inhibition cannot be used to investigate gene functions in the aquatic symbiosis between *Bodo saltans* and its bacterial symbiont. Our work stresses the importance of the development of alternative protocols to study the biology of both the microeukaryote and the associated bacterium.

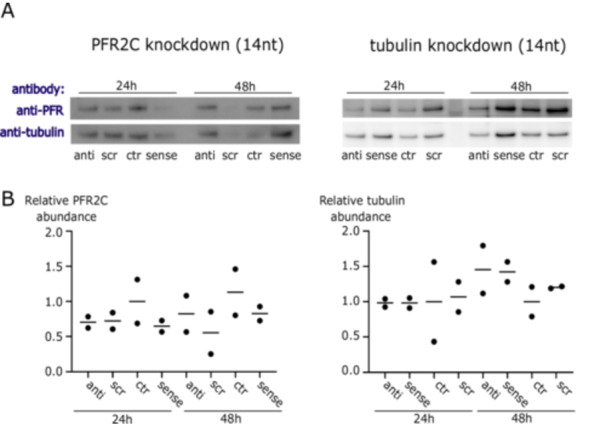

## 1 Introduction

Dependent symbioses—where a host requires a symbiont for function—are common in nature. They also present therapeutic opportunities in some cases, as in filarial diseases, where targeting of the symbiont enables sterilization of the host and ultimately a novel treatment strategy (Taylor et al. 2005; Landmann et al. 2011). More widely, many blood‐feeding vectors and agriculturally important phloem‐feeding insects rely on symbiont presence, such that understanding the basis of dependence is important in health and food security (Lai et al. 1994; Douglas 1998; Hosokawa et al. 2007, 2010; Hirota et al. 2017; Heddi et al. 1999; Balmand et al. 2013). However, the symbionts (and sometimes the host) are commonly refractory to functional analysis, inhibiting our capacity to understand the interplays resulting in dependence.

Microeukaryotic hosts present an opportunity for understanding symbiotic relationships. A broad range of symbiotic microbes inhabit single‐cell eukaryotes, with diverse impacts on their host. For example, in the *Paramecium–Chlorella* symbiosis, the algal endosymbiont provides photosynthetic capability to the microeukaryote host (Hoshina and Kusuoka 2016). Additionally, the stability of this symbiosis is ensured by the “penalty system” acting on the host upon the death of the symbiont (Jenkins et al. 2021). Killing of the symbiont releases its mRNA with a high level of sequence identity to host transcripts. These mRNAs are then processed by the host RNAi machinery, resulting in the knockdown of endogenous host gene expression, which is detrimental to the host. Therefore, RNA–RNA interactions can be crucial in maintaining symbiosis (Jenkins et al. 2021). The simplicity of microeukaryote culture, their susceptibility to RNA‐level manipulation of gene expression, and the possibility of delivery of small molecules to cells through the culture medium make them a potentially useful tool in enabling functional analysis. However, the development of these tools for aquatic symbioses is in its infancy.

To this end, we wished to establish tools for functional analysis in the interaction between the free‐living flagellated kinetoplastid *Bodo saltans* and its intracellular bacterium, C*andidatus Bodocaedibacter vickermanii* (Cbv), a member of Rickettsiales (Midha et al. 2021). *B. saltans* is a unicellular eukaryote hosting Cbv (Midha et al. 2021). It is a heterotroph that feeds on extracellular bacteria and is found in freshwater and marine environments (Mitchell et al. 1988). Cbv appears to be a permanent inhabitant of *B. saltans* cells (Midha et al. 2021). An obligate symbiosis between *B. saltans* and Cbv has been postulated, based on the observation that antibiotic clearance of the bacterium kills the eukaryote as well (Midha et al. 2021). Genomic analysis of Cbv revealed the presence of the Bacterial Polymorphic Toxin Systems with a putative role in symbiosis maintenance. It has been hypothesized that the removal of the symbiont with antibiotics would stop the production of the antitoxin and render *B. saltans* cells defenseless in the presence of a longer‐lived bacterial toxin.

To study *B. saltans*–Cbv symbiosis in more detail, we need tools for simultaneous manipulation of gene expression in host and symbiont. Antisense inhibition is a good candidate for a universal protocol working across different kingdoms. It has been achieved multiple times in trypanosomes closely related to *B. saltans*. In *Trypanosoma cruzi*, Surface Glycoprotein gp90 was blocked by the antisense approach, using a phosphorothioate oligonucleotide based on a sequence of the *gp90* coding strand (Málaga and Yoshida 2001). Similarly, the expression of Calcineurin B of this parasite has also been knocked down to show that this protein is involved in cell invasion (Araya et al. 2008). Finally, *T. cruzi*, inositol 1,4,5‐trisphosphate receptor has also been inhibited by the antisense oligonucleotide treatment (Hashimoto et al. 2014).

Antisense inhibition can also be applied to bacteria (Good and Nielsen 1998; Good 2002), including *Rickettsia* and *Ehrlichia* (Pelc et al. 2015; Sharma et al. 2017), which are closely related to Cbv. Synthetic peptide nucleic acid (PNA) molecules targeting mRNA for *rOmpB* (Rickettsial outer‐membrane protein B) in *Rickettsia typhi* and *rickA* (Arp2/3 complex activator) in *Rickettsia montanensis* have successfully induced antisense inhibition of these genes, confirming their importance for infection (Pelc et al. 2015). Additionally, PNAs have been used to knock down *Ehrlichia* translocated factor‐1 (Etf‐1), which induces Rab5‐regulated autophagy to provide host cytosolic nutrients required for *Ehrlichia chaffeensis* proliferation. This knockdown reduced the bacteria's ability to infect host cells (Sharma et al. 2017). Importantly, the intracellular localization of Rickettsiales often requires carriers enabling antisense molecules to move across membranes. Molecular carriers have been successfully employed to deliver specific and lasting antisense inhibition to intracellular *Chlamydia* (Mishra et al. 2012) and DNA to another Cbv relative—*Anaplasma (*Oki et al. 2015 *)*.

As the development of genetic tools for the host *B. saltans* is beginning to gain traction (Faktorová et al. 2020; Gomaa et al. 2022), we attempted to develop knockdown protocols for both host and symbiont gene expression. We show that fluorescently labeled antisense molecules reach the *B. saltans* cytoplasm as well as intracellularly localized bacteria. However, antisense inhibition was not achieved. Upon antisense compound treatment, *B. saltans* cells proliferate excessively, changing the protein composition of the cells. We hypothesize that *B. saltans* (or the bacteria it feeds upon) consume antisense molecules and experience a growth boost by a mechanism unrelated to antisense inhibition.

## 2 Results

### 2.1 Identifying Carriers Able to Deliver Antisense Molecules to *B. saltans* and Its Intracellular Symbiont

Initially, we determined the best delivery protocol to introduce antisense molecules into *B. saltans* and Cbv cells. We treated *B. saltans* cells with fluorophore‐conjugated synthetic PNAs, as these molecules have been most thoroughly tested in intracellular bacteria. For intracellular delivery, we used two different protocols: incubation of *B. saltans* cells for 24 h and electroporation of *B. saltans* with 50 µM PNA molecule PNA00218_TMR (Table 1).

**Table 1:** Oligonucleotide analogs designed and tested for *B. saltans* and its symbiont.

Name	Sequence	Backbone and modifications	Source
PNA00218_TMR	GATCCAATGC‐Lys(TMR)	PNA	Panagene
Chol‐OO‐PNA00218_TMR	Chol‐OO‐GATCCAATGC‐Lys(TMR)	PNA, cholesteryl hemisuccinate‐conjugated	Panagene
R8‐PNA00218_TMR	H‐RRRRRRRR‐GATCCAATGC‐ Lys(TMR)	PNA, R8‐conjugated	Panagene
00218_PS_TAMRA	G*AT* CCA ATG CA*A ACT GAA AT*T TAT GAA TTT* ATG* C‐TMR	Phosphorothioate backbone bases are marked with “*”	IDT
00218‐6‐2ome‐TAMRA	mGmAmU CCA ATG CAA mACT GAA ATT mUAT GAA TTT mAmUmG mCmG‐TMR	2'Ome RNA bases are marked with “m”	IDT
00218_anti	GATCCAATGCAA	*PNA*	Panagene
00218_sense	TTGCATTGGATC	*PNA*	Panagene
Tubulin_anti_14nt	CGCGAGATCGTTTC	*PNA*	Panagene
Tubulin_anti_20nt	CGCGAGATCGTTTCCATCCA	*PNA*	Panagene
Tubulin_sense_20nt	TGGATGGAAACGATCTCGCG	*PNA*	Panagene
Tubulin_sense_14nt	GAAACGATCTCGCG	*PNA*	Panagene
PFR2C_anti_14nt	AACATGGCCGACCA	*PNA*	Panagene
PFR2C_anti_20nt	AACATGGCCGACCAACAACC	*PNA*	Panagene
PFR2C_sense_14nt	TGGTCGGCCATGTT	*PNA*	Panagene
PFR2C_sense_20nt	GGTTGTTGGTCGGCCATGTT	*PNA*	Panagene
PFR3_anti_14nt	ATCATGCCCGAGGA	*PNA*	Panagene
PFR3_anti_20nt	AACAACATCATGCCCGAGGA	*PNA*	Panagene
PFR3_sense_14nt	TCCTCGGGCATGAT	*PNA*	Panagene
PFR3_sense_20nt	TCCTCGGGCATGATGTTGTT	*PNA*	Panagene
Scr‐12nt	GTAAACATCACG	*PNA*	Panagene
Scr‐14nt	ATGACCGTCTGCTG	*PNA*	Panagene
Scr‐20nt	ACAGCCAGAACTACCCAGAC	*PNA*	Panagene

**Figure 1:**
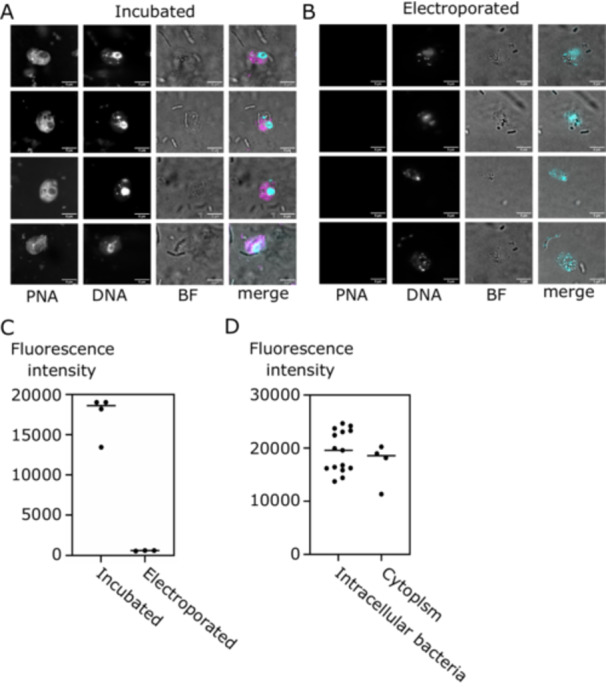
Incubation is more efficient than electroporation in delivering fluorescent PNAs to *B. saltans* and Cbv. Fluorescent images of *B. saltans* (A) incubated or (B) electroporated with fluorescently labeled antisense molecules. In merged images, DNA is in cyan and TMR in magenta. (C) Quantification of fluorescence intensity in the cytoplasm of *B. saltans* cells depicted in Figure 1A,B. Only images acquired with the same settings were used for quantification. (D) Quantification of fluorescence intensity in the intracellular bacteria and the cytoplasm (excluding the bacteria) in *B. saltans* incubated with antisense molecules depicted in Figure 1A. Imaging parameters are in Table S1.

We imaged *B. saltans* cells after 24 h to ascertain localization of the fluorescent antisense molecule within hosts and symbionts (Figure 1A). In our experiment, incubation resulted in much higher fluorescence intensity within the *B. saltans* cells compared to electroporation (Figures 1A,B and S1). This impact might be related to the fact that incubated cells are in contact with antisense molecules for 24 h, while the electroporation protocol allows for just a very brief contact with antisense molecules (during electroporation).

Next, we determined whether PNA00218_TMR can enter bacterial cells within the *B. saltans* cytoplasm. To this end, we quantified the fluorescence intensity within the intracellular bacteria of *B. saltans* and compared it to the fluorescence intensity of the cell cytoplasm (excluding bacteria and nucleus). Intracellular bacteria had slightly higher average fluorescence intensity (19,383 AU vs. 17,192 AU) than the surrounding cell cytoplasm, meaning that antisense molecules can enter Cbv cells but do not disproportionately accumulate within the symbiont.

In the process of optimizing the delivery, we also assessed other antisense molecules chemistries, including PNAs attached to peptide and lipid carriers, 2'ome RNAs, and phosphothiorate oligos (Table 1). However, they all consistently yielded weaker intracellular fluorescence by both incubation and electroporation (Figure S2) in *B. saltans* cells. Incubation of *B. saltans* with PNAs became our protocol of choice for subsequent *B. saltans* and Cbv targeting experiments.

### 2.2 Antisense Inhibition of Host and Symbiont Genes

To achieve antisense inhibition of *B. saltans* and intracellular symbiont, we designed antisense oligonucleotides spanning the start codon and ribosome binding site of three host and two symbiont‐encoded genes (Good 2002). The choice was dictated by the availability of specific antibodies for the host, while for the symbiont, we targeted proteins with readily predictable function. In *B. saltans*, our targets were PFR2C and PFR3 (paraflagellar rod protein isoforms putatively involved in *B. saltans* feeding) (Gomaa et al. 2022) and tubulin. For Cbv, we used an antitoxin from one of the putative toxin–antitoxin operons (CPBP_00218 gene) and a putative cell division protein ftsZ. Bacterial antisense targeting is best achieved with 10‐nucleotide‐long antisense molecules (Good 2002). For *B. saltans*, genome complexity precludes the design of such short yet specific antisense molecules. Thus, we focused on 14 and 20 nucleotide antisense molecules. For all experiments, we have also used scrambled nontargeting oligos as a control.

**Figure 2:**
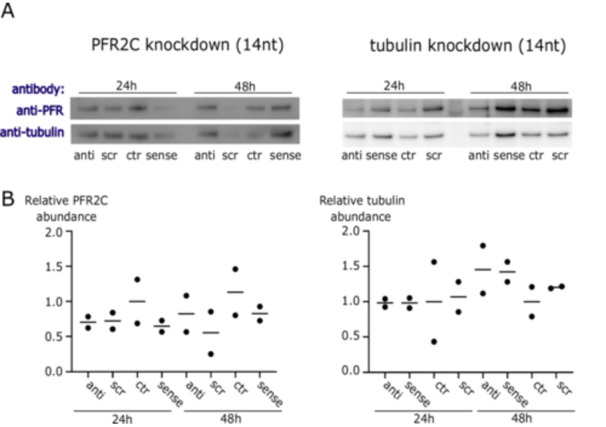
Antisense inhibition is not effective in *B. saltans*. (A) Western blot with antibodies against PFR (L8C4) and tubulin (KMX‐1) on whole *B. saltans* protein extract*. B. saltans* cells were treated with antisense molecules against PFR2C+PFR3 (the PFR isoforms recognized by the L8C4 antibody) and tubulin. Antisense, sense, scrambled, and no antisense oligonucleotides were incubated with *B. saltans* cells for either 24 or 48 h. (B) Quantitative PCR for transcript abundance of genes targeted by antisense inhibition against PFR2C+PFR3 (left) or tubulin (right).

First, we treated *B. saltans* cells with antisense PNAs for 24 and 48 h designed to silence PFR2C and PFR3 (together for Western blot, as our antibody detects both protein isoforms, and separately for qPCR as we can detect each mRNA/cDNA separately) and tubulin (Figure 2). We did not find evidence of antisense inhibition either by Western blot or by qPCR against targeted transcripts. Although protein samples were quantified and normalized for input, we observed stochasticity in PFR and tubulin protein levels. Therefore, we have additionally confirmed that our Western blots were quantitative (Figure S3) and that the differences in protein levels visible in Figure 2A reflect *B. saltans* biology.

**Figure 3:**
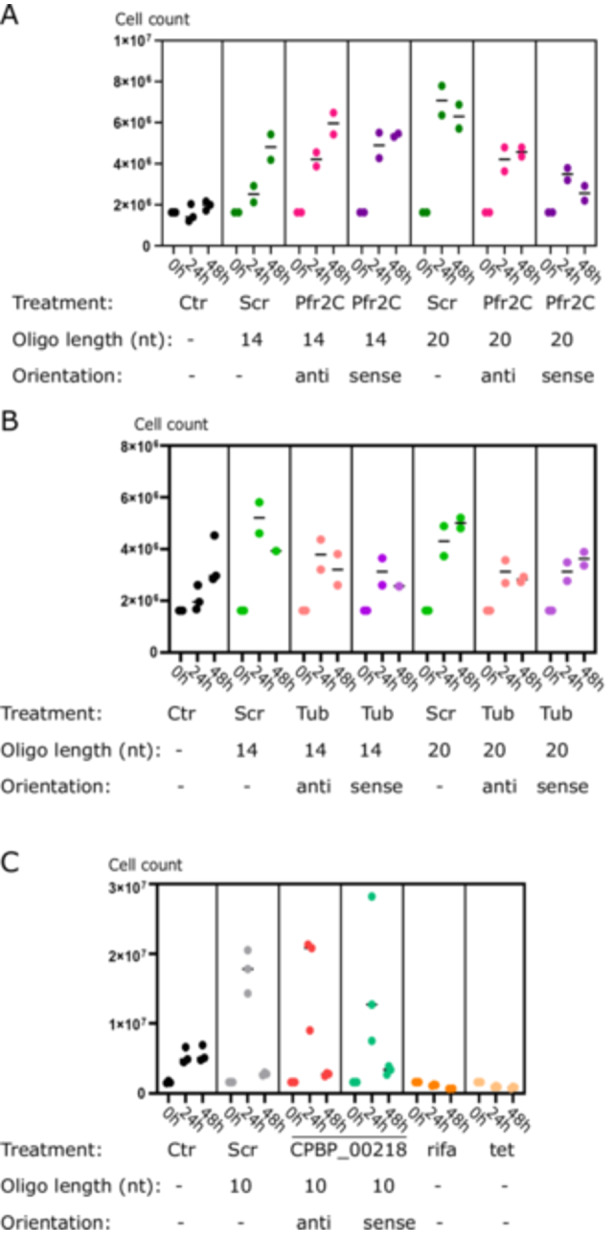
*B. saltans* proliferates massively when exposed to peptide nucleic acid molecules. Cell counts of *B. saltans* treated with antisense molecules against (A) PFR2C+PFR3, (B) tubulin, and (C) Cbv gene CPBP_00218. In all cases, *B. saltans* exposed to peptide nucleic acids reaches higher cell numbers. Rifa and tet are rifampicin and tetracycline, the controls used to see loss of *B. saltans* viability, which we expected with the CPBP_00218 gene knockdown.

Due to the lack of specific antisense effect on *B. saltans* cells and the stochastic *B. saltans* protein abundance (especially in loading controls in the above experiments [Figure 2]), we checked whether the cell numbers of *B. saltans* were altered upon antisense PNA treatment (Figure 3). To our surprise, *B. saltans* proliferated massively in culture upon antisense PNA addition. Although the strength of the effect depended on the oligonucleotide length and sequence, it was universal for the *B. saltans* and Cbv‐targeting and nontargeting PNAs.

## 3 Discussion

The study of obligate symbioses presents significant challenges for genetic analysis, particularly when dealing with non‐model species and unculturable symbionts. We focused on the kinetoplastid host, *B. saltans*, and its bacterial symbiont, Cbv—an association that exemplifies these challenges. Our aim was to develop tools for the analysis of gene function within this symbiosis using antisense inhibition. Our results demonstrated that antisense inhibition is not feasible in *B. saltans* and its symbiont under the conditions tested. Despite the successful delivery of fluorescently labeled antisense molecules into the *B. saltans* cells and into their intracellular bacteria, we did not achieve the anticipated knockdown of gene expression. Instead, we observed a surprising proliferation of *B. saltans* upon treatment with the antisense constructs. We have two potential explanations for this effect. First, that *B. saltans* itself may derive nutrition from the antisense molecules. Second, *Klebsiella*—the prey of *B. saltans*—may derive nutrition, indirectly fueling *B. saltans* proliferation.

PNAs are synthetic mimics of DNA in which the deoxyribose phosphate backbone is replaced by a pseudo‐peptide polymer to which the nucleobases (purines and pyrimidines) are linked (Good and Nielsen 1998; Nielsen et al. 1991). Repetitive units of *N*‐(2‐aminoethyl) glycine constituting the backbone of PNAs have been shown to be from 30× to 1000× more resistant to different proteases than the peptides of the same length. However, they are not entirely resistant to proteolysis (Demidov et al. 1994). *B. saltans* has a particularly high number of genes involved in the breakdown of peptides, and bacterial amino acids are the major source of energy, carbon, and nitrogen for this eukaryote (Jackson et al. 2016). Additionally, *B. saltans* is a purine auxotroph (Jackson et al. 2016). Therefore, PNAs in the medium might constitute an easy source of essential nutrients for *B. saltans*, promoting its growth.

Moreover, *B. saltans* feeds on bacteria—*K. pneumoniae*—in our laboratory cultures. These bacteria do not seem to quench the antisense molecules (Figure 1A,B notes the extracellular bacteria in the bright‐field images). Nevertheless, they might be excreting proteases into the medium (Brinkworth et al. 2015), impeding the delivery of full‐length PNAs to the *B. saltans* and Cbv, even when the fluorophore is internalized. Additional nutrients produced in the process might feed *K. pneumoniae* or *B. saltans*, inducing the proliferation of the latter.

The inability to achieve antisense inhibition in *B. saltans* and Cbv suggests that this method, while effective in other systems such as trypanosomes and Cbv‐related bacteria (Málaga and Yoshida 2001; Araya et al. 2008; Hashimoto et al. 2014; Pelc et al. 2015; Hyrup and Nielsen 1996), may not be universally applicable across different species and symbiotic contexts. The unique metabolic and cellular processes of *B. saltans* or its microbiome might interfere with the antisense mechanism, necessitating alternative approaches for manipulation. Other gene targeting techniques, such as homologous recombination and CRISPR/Cas9, are currently being established for *B. saltans* (Faktorová et al. 2020; Gomaa et al. 2022), and further optimization might make them available for its intracellular symbionts as well. In the future, they will enable thorough investigation of the molecular basis of the symbiotic relationship between *B. saltans* and Cbv, including the role of the mysterious Bacterial Polymorphic Toxin Systems in maintaining this obligate symbiosis.

Although our attempt to use antisense inhibition in *B. saltans* and its symbiont Cbv was unsuccessful, our study provides important insights and protocols that contribute to the broader effort of understanding and manipulating gene function in complex symbiotic systems. We developed electroporation, incubation, live imaging, image analysis, and Western blot analysis protocols and identified a set of antibodies cross‐reactive with *B. saltans*. These will strengthen our future efforts to identify genetic underpinnings of the obligate *B. saltans–*Cbv symbiosis.

## 4 Materials and Methods

### 4.1 *B. saltans* Culture

*B. saltans* was cultured in a cerophyl‐based medium enriched with 3.5 mM sodium phosphate dibasic (Na_2_HPO_4_) (Gomaa et al. 2022). Cultures were incubated at 22°C in T25 tissue culture flasks containing 20 mL of media bacterized with *K. pneumoniae* subsp. *pneumoniae* (ATCC 700831). 3–4‐day‐old cultures were used for experiments.

### 4.2 Antisense Molecules

The intended mode of action of our antisense constructs was physical protection of the targeted mRNA from being subjected to posttranscriptional processing and/or translation of proteins. PNAs are synthetic DNA mimics in which the deoxyribose phosphate backbone is replaced by a pseudo‐peptide polymer (*N*‐(2‐aminoethyl)glycine units) to which the unmodified nucleobases are linked (Nielsen et al. 1991). PNAs hybridize with complementary DNA or RNA with high affinity and specificity (Hyrup and Nielsen 1996). Although the most recent application of PNA is as hybridization probes in fluorescent in situ hybridization (FISH) experiments, their best‐described use is antisense inhibition of target genes (Good and Nielsen 1998; Pellestor and Paulasova 2004; Koppelhus and Nielsen 2003). PNAs can interfere with transcription and translation of genes by tightly binding to DNA or mRNA and are thought to act as blocking agents. In vitro studies have demonstrated PNA binding to complementary DNA, effectively inhibiting transcriptional elongation and preventing the binding of transcription factors (Boffa et al. 1996; Nielsen et al. 1994). For antisense design, we followed a previously described strategy (Dryselius et al. 2003), with short oligomers against bacterial transcripts and longer ones for eukaryotic gene blocking (Good and Nielsen 1998). For phosphorothioate antisenses targeting *B. saltans* genes, we followed the design of antisense oligos for the related trypanosomatid *T. cruzi* (Málaga and Yoshida 2001). We used phosphorothioate antisenses and 2'‐O‐Methyl (2'OMe) RNA‐DNA design with modified nucleotides at the extremities and inside the sequence to protect the construct from both exo‐ and endonucleases (Lohman 2024). Antisense PNAs were synthesized by Panagene, South Korea. Other oligonucleotides were synthesized by IDT. All antisense oligonucleotides were ordered with HPLC purification and > 90% purity and subjected to the manufacturer's quality checks.

### 4.3 Incubation of *B. saltans* Cells With Antisense Molecules

*B. saltans* cultures were filtered through 100 and 8 µm filters. Cells were harvested by centrifugation at 1200 × g for 12 min at 19°C, washed with 10–15 mL sterile filtered (SF) 1× PBS, and cells re‐suspended in 5 mL SF 1× PBS. Single treatment cell aliquots containing 5 × 10^5^ cells were centrifuged again, and PBS was replaced with SF cerophyll medium. Antisense molecules were added in a final concentration of 50 µM. After 24 h and 48 h, *B. saltans* cells were harvested for Western blot and qPCR. The detailed protocol can be found here: dx.doi.org/10.17504/protocols.io.6qpvr8pqblmk/v1.

### 4.4 Electroporation of Fluorescent Antisense Molecules Into *B. saltans* and Live Imaging

We electroporated antisense molecules into *B. saltans* cells according to the published protocol (https://www.protocols.io/view/electroporation-of-fluorescent-antisense-molecules-e6nvwk8e2vmk/v1). To prepare *B. saltans* cells for electroporation, the culture was first filtered through 100 µm and 8 µm filters. The cells were then harvested by centrifugation at 1200 × g for 12 min at 19°C. Following this, the cells were washed with 10 mL PBS and centrifuged again under the same conditions. The cells were resuspended in 5 mL PBS, counted using a hemacytometer, and the volume containing 5 × 10^5^ cells was taken as recommended for the Neon transfection kit (Thermo Fisher Scientific) with a 10 µL tip. The cells were centrifuged once more at 1200 × g for 12 min at 19°C. After removing the PBS, the cells were resuspended in electroporation buffer. An antisense molecule was then added to the *B. saltans* cells at a final concentration of 50 µM and mixed by pipetting. Finally, the mixture was aspirated into a Neon pipette and electroporated with a single pulse at 1800 V with a 10 ms pulse width.

The antisense molecule concentration was chosen based on our preliminary experiments showing that the concentration used in the literature (20 µM) was not effective in our system (Málaga and Yoshida 2001; Kurupati et al. 2007). Given our experimental design, this was also the highest achievable antisense molecule concentration, in the range of concentrations used for other experiments involving trypanosomatids (Hashimoto et al. 2014; Málaga and Yoshida 2001) and 10× to 100× more than in previous experiments showing antisense inhibition in bacteria (Good and Nielsen 1998).

### 4.5 Imaging

To image *B. saltans* cells, we mixed, incubated, or electroporated cells with 1% low melting temperature agarose (Thermo Fisher Scientific) in a 1:1 ratio and let it set for a few seconds in a well of a 96‐well plate. We stained DNA with Hoechst 33342 (Thermo Fisher Scientific) diluted 1:2000 in PBS by adding the solution to the agarose‐embedded *B. saltans* and incubating it for 10 min at room temperature (RT). We washed the agarose with PBS (2 × 5 min) at RT. We removed the agarose from the well with clean forceps, placed it on a microscope slide, added a drop of mounting medium (Vectashield, Vector Laboratories), and flattened the agarose using the coverslip. We proceeded with confocal imaging at the Centre for Cell Imaging using an LSM 880 Laser Scanning Confocal (Zeiss) equipped with a 63× (1.4 NA) objective, and parameters listed in Table S1. Because the bright‐field images were used to localize the *B*. saltans and for general reference only, the acquisition and display parameters were adjusted individually for each image. Images were analyzed using custom Fiji (Schindelin et al. 2012) Script provided by Marie Held at the Centre for Cell Imaging, University of Liverpool. The protocol for analysis can be found here: dx.doi.org/10.17504/protocols.io.6qpvr8pqblmk/v1, and the script for automation of analysis can be found here: https://github.com/Marien-kaefer/General_Fiji_macros/tree/main/BodoSaltans.

### 4.6 Western Blot

We performed Western blots according to the published protocol (https://www.protocols.io/view/protein-extraction-quantification-and-western-blot-5qpvobqodl4o/v2). Cells were harvested as above, and protein was extracted according to Newton et al. (2015). Subsequently, protein was quantified with Pierce BCA Protein Assay Kit (Thermo Fisher Scientific) according to the manufacturer's instructions. All concentrations were normalized to those of the most diluted samples. Samples were reduced, separated on polyacrylamide gels (Bolt Bis‐Tris Plus, Thermo Fisher Scientific), and then transferred to PVDF membranes using a mini blot module (Thermo Fisher Scientific). The membranes were blocked for 30 min at RT in 5% skimmed milk diluted in wash buffer (PBS with 0.1% Tween 20). Subsequently, the membranes were incubated overnight at 4°C with primary antibodies (Thermo Fisher Scientific, diluted 1:100 in 5% milk in wash buffer, Table 2). After incubation, the membranes were washed three times for 10 min each with wash buffer. They were then incubated for 1 h at RT with horseradish peroxidase (HRP)‐labeled secondary antibodies (diluted 1:5000 in 5% milk in wash buffer). The membranes were washed again three times for 10 min each with wash buffer. Finally, the signal was developed using an HRP substrate (e.g., Immobilon western kit, Merck) and scanned for chemiluminescent signal using an imaging system (e.g., ImageQuant LAS4000 or Bio‐Rad Chemidoc). The exposure time was chosen for each membrane separately to the highest value possible while avoiding saturated pixels, and results were saved as 16‐bit images.

**Table 2:** Antibodies tested for cross‐reactivity with *B. saltans*.

Name	Target	Source	Cross‐reactive with *B. saltans*
BBA4	Basal body	Jack Sunter/Keith Gull (Kohl et al. 1999; Birkett et al. 1985)	No
ROD1	Paraflagellar rod	Jack Sunter/Keith Gull (Kohl et al. 1999; Birkett et al. 1985)	No
L8C4	Paraflagellar rod	Jack Sunter/Keith Gull (Kohl et al. 1999; Birkett et al. 1985)	Yes
L3B2	Flagellum attachment zone	Jack Sunter/Keith Gull (Kohl et al. 1999; Birkett et al. 1985)	No
KMX‐1	Tubulin	Jack Sunter/Keith Gull (Kohl et al. 1999; Birkett et al. 1985)	Yes
D6A8	β‐actin	Cell Signaling Technology	Yes

### 4.7 RNA Extraction, cDNA Synthesis, and qPCR

*B. saltans* cells were homogenized in Trizol Reagent (Invitrogen) and RNA was extracted using a Direct‐zol RNA MiniPrep kit (Zymo Research), including a DNAse digestion step according to the manufacturer's instructions. RNA was eluted in 25 µL DNase/RNase‐Free Water (Zymo Research), and the concentration was determined using a NanoDrop Spectrophotometer. cDNA was prepared from 1 µg of total RNA using Random Primers and M‐MLV Reverse Transcriptase (both Promega). Primers were allowed to bind to template RNA for 5 min at 70°C, followed by 25°C for 10 min before M‐MLV was added and incubated at 37°C for 60 min and then 80°C for 10 min.

Real‐time qPCRs were carried out in a LightCycler 480 Instrument II (Roche) with the Power SYBR Green PCR Master Mix (ThermoFisher Scientific). Each reaction contained 6 µL of SYBR Green PCR Master Mix, 0.5 µL of each primer solution at 3.6 µM, and 5 µL of diluted DNA. Each plate contained three technical replicates of every sample for each set of primers. Primers used are listed in Table 3. Relative expression levels were calculated by the Pfaffl method (Pfaffl 2001), with PFR2C relative to tubulin and tubulin relative to actin.

**Table 3:** Oligonucleotide primers used for qPCR.

Oligo name	Oligo sequence (5′ ‐> 3′)
qTubulin_fwd	CTTCCAGATCTCCCACTCCC
qTubulin_rev	TCATCATGATACGGTCGGGG
qPFR2C_fwd	AGTACCAGCAGTTCCTCGAC
qPFR2C_rev	GCTCCTCGATGATGCCAATG
qAct_fwd	CTCGTACCAAATCCGTGCAG
qAct_rev	AACAACATTCCACGACGAGC

## Author Contributions

**Mastaneh Ahrar:** investigation, methodology, visualization, data curation, validation, writing – review and editing. **Lorna Glenn:** investigation, writing – review and editing, data curation, validation. **Marie Held:** methodology, software, writing – review and editing, visualization. **Andrew Jackson:** conceptualization, funding acquisition, writing – review and editing. **Krzysztof Kus:** methodology, software, writing – review and editing. **Gregory D. D. Hurst:** supervision, conceptualization, funding acquisition, resources, writing – original draft, project administration, writing – review and editing. **Ewa Chrostek:** conceptualization, investigation, funding acquisition, writing – original draft, methodology, data curation, supervision, formal analysis, visualization, validation, writing – review and editing.

## Ethics Statement

The authors have nothing to report.

## Conflicts of Interest

The authors declare that the research was conducted in the absence of any commercial or financial relationships that could be construed as a potential conflicts of interest.

## Supporting information


**Figure S1. Incubation is more efficient than electroporation in delivering fluorescent PNAs** ***B***. ***saltans*** **and Cbv.** Fluorescent images of *B. saltans* A) incubated or B) electroporated with fluorescently labelled antisense molecules. This image was acquired with different TMR channel settings than Fig. 1. Imaging parameters are in Table S1.

**Figure S2. Untagged PNAs incubated with** ***B***. ***saltans*** **are the most effective in entering microeukaryote cells**. A) PNAs, PNAs tagged with a cell penetrating peptide R8 or cholesteryl hemisuccinate, and 2'ome RNA were incubated with the cells. B) Phosphorothioate oligo was electroporated into *B. saltans* and imaged using 3x zoom. This image was cropped for display but cannot be directly compared with the other images. C) No antisense molecule control to visualize potential *B. saltans* autofluorescence. In merged images DNA is in cyan, TMR in magenta. Sequences and modifications of each of the molecules are listed in Table 1. Imaging parameters are in Table S1.

**Figure S3. Western blot is a quantitative method for** ***B***. ***saltans***. Western blot with antibodies against PFR (L8C4) and tubulin (KMX‐1) were used to probe a membrane with different quantities of whole *B. saltans* protein extract.

Table S1. Imaging parameters for figures included in the manuscript.

## Data Availability

The data that support the findings of this study are openly available in Figshare at https://figshare.com/, reference number 10.6084/m9. figshare.26394475.v1. All raw data can be accessed on Figshare (https://figshare.com): 10.6084/m9. figshare.26394475.v1.
