# Supplementary figures and images for: Development of Antisense Tools to Study Bodo saltans and Its Intracellular Symbiont
Source: Microbiologyopen. 2025 Apr 23;14(2):e70018. doi: 10.1002/mbo3.70018 (PMC12018706; doi:10.1002/mbo3.70018)

A

Incubated

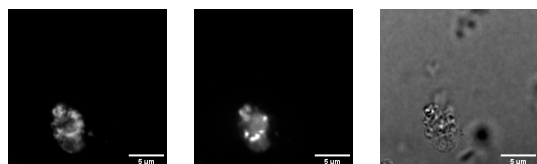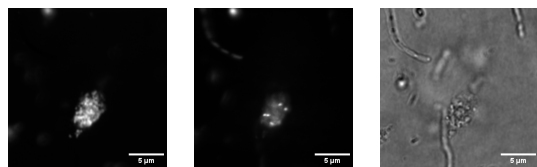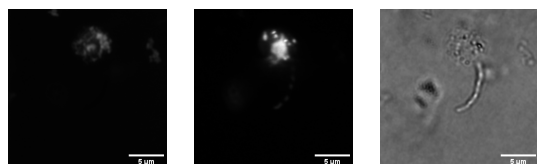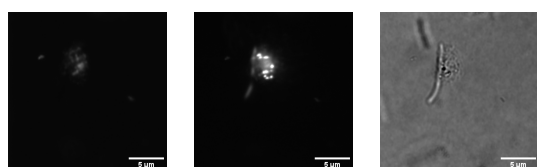

PNA

DNA

BF

B

Electroporated

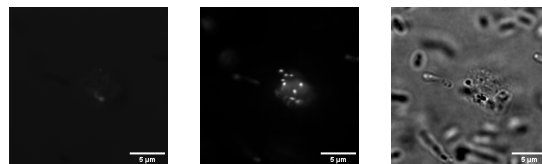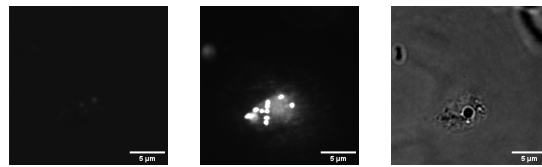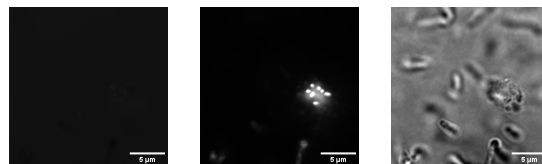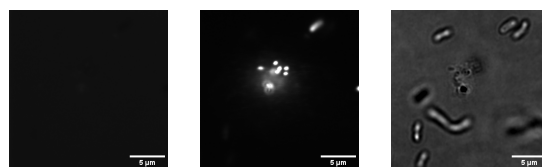

PNA

DNA

BF

Supplement: Supplementary file 1 — Figure S1. Incubation is more efficient than electroporation in delivering fluorescent PNAs B . saltans and Cbv. Fluorescent images of B. saltans A) incubated or B) electroporated with fluorescently labelled antisense molecules. This image was acquired with different TMR channel settings than Fig. 1. Imaging parameters are in Table S1. [file MBO3-14-e70018-s003.pdf]

A

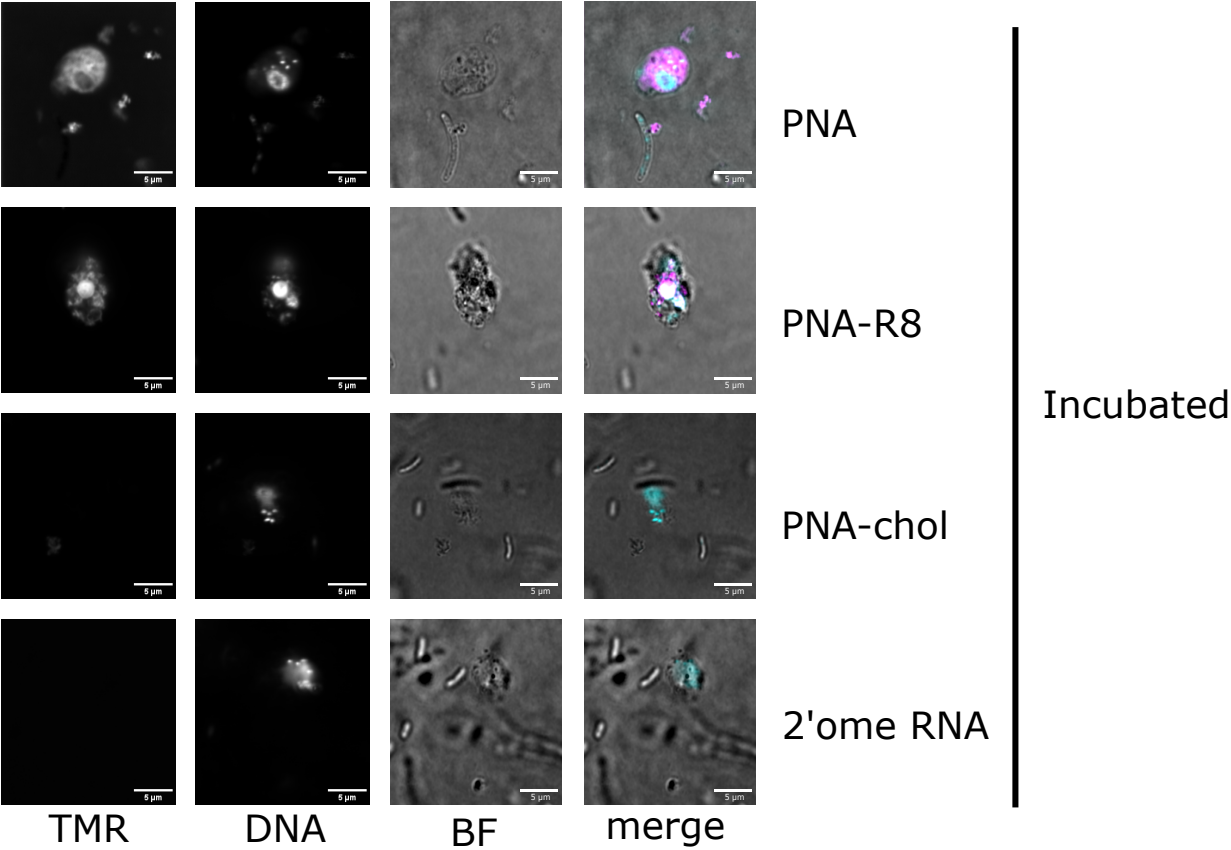

B

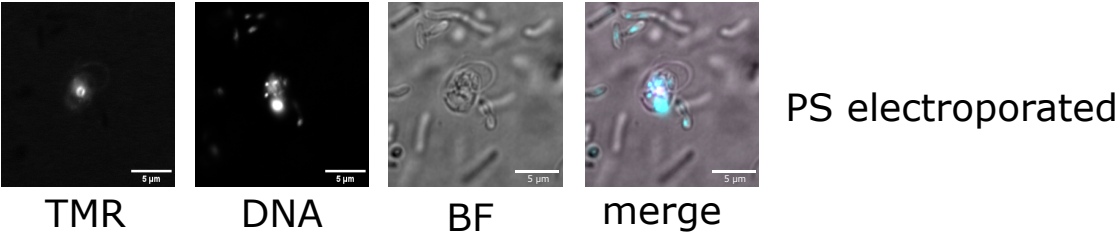

C

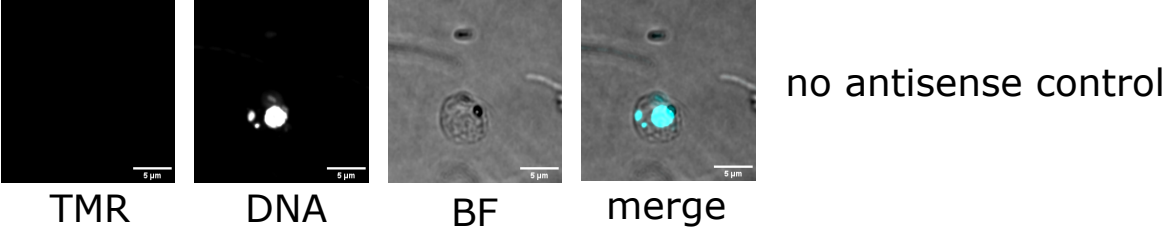

Supplement: Supplementary file 2 — Figure S2. Untagged PNAs incubated with B . saltans are the most effective in entering microeukaryote cells. A) PNAs, PNAs tagged with a cell penetrating peptide R8 or cholesteryl hemisuccinate, and 2'ome RNA were incubated with the cells. B) Phosphorothioate oligo was electroporated into B. saltans and imaged using 3x zoom. This image was cropped for display but cannot be directly compared with the other images. C) No antisense molecule control to visualize potential B. saltans autofluorescence. In merged images DNA is in cyan, TMR in magenta. Sequences and modifications of each of the molecules are listed in Table 1. Imaging parameters are in Table S1. [file MBO3-14-e70018-s002.pdf]

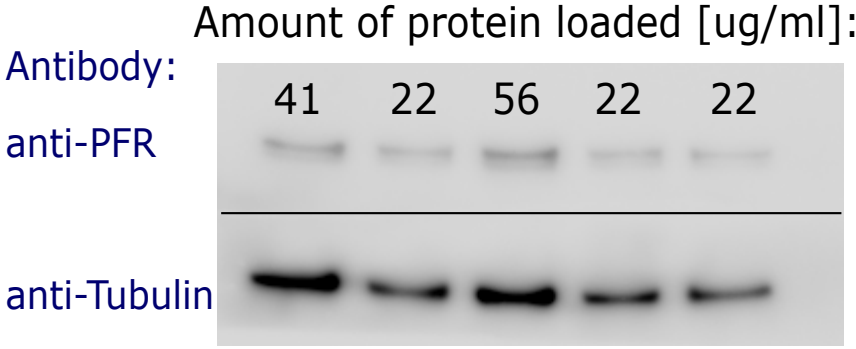

Supplement: Supplementary file 3 — Figure S3. Western blot is a quantitative method for B . saltans . Western blot with antibodies against PFR (L8C4) and tubulin (KMX‐1) were used to probe a membrane with different quantities of whole B. saltans protein extract. [file MBO3-14-e70018-s004.pdf]
